# Identification Bracelet Precipitated Acute Compartment Syndrome during Intravenous Infusion in an Obtunded Patient

**DOI:** 10.1155/2016/8506357

**Published:** 2016-01-19

**Authors:** Wahib Zafar, Benjamin Chaucer, Suleyman Felek, Edward L. Arsura, Jay Nfonoyim

**Affiliations:** ^1^Department of Medicine, Richmond University Medical Center, Staten Island, NY 10301, USA; ^2^St. Georges University School of Medicine, 3500 Sunrise Highway, Great River, NY 11739, USA

## Abstract

Acute compartment syndrome is a serious condition requiring immediate medical care. A lack of urgent medical treatment can result in serious complications such as loss of function and even amputation. While the pathophysiology of acute compartment syndrome is well understood, numerous potential causes are still being discovered. A rare cause of acute compartment syndrome is IV infiltration. We present a case of acute compartment syndrome resulting from intravenous infusion due to proximal placement of a patient identification bracelet. We conclude that both routine evaluation for IV infiltration and proximal placement of IV lines are essential for prevention of acute compartment syndrome.

## 1. Introduction

Acute compartment syndrome (ACS) is the compromise of tissue due to increased pressure in the fascial spaces. The increase in pressure in the closed fascial space deteriorates capillary perfusion and results in nerve and muscle damage and tissue necrosis [1–4]. Acute compartment syndrome is a serious limb threatening condition; if it is not recognized and treated early, consequences of ACS will be not only permanent loss of function but also limb loss [2]. Acute compartment syndrome can result from a variety of sources. Common causes include closed fractures, crush injuries, increased capillary permeability, and burns [1, 3]. Intravenous (IV) infiltration is a common entity seen in hospitals and often results in minor complications such as minimal swelling or erythema. In severe cases, increase in tissue pressures may lead to ACS. This is seen mainly in large fluid or automated infusions [1, 4, 5]. In our patient the location of a patient identification bracelet proximal to an IV infusion line leads to a rare presentation of acute compartment syndrome.

## 2. Case Report

**Figure 1:**
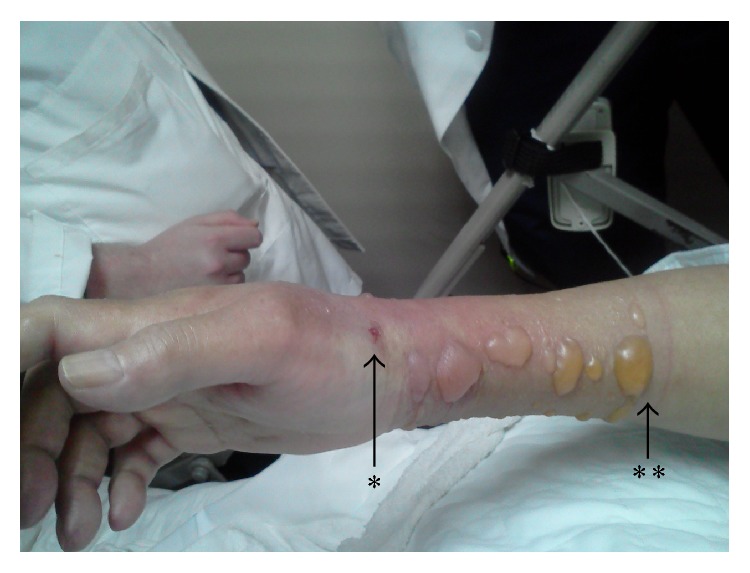
The picture of the patient's arm just after infiltration was identified. ^*∗*^IV line site, ^*∗∗*^ID bracelet site.

An 83-year-old female was brought in by ambulance due to rapid cognitive decline leading to decreased oral intake. Her past medical history includes Parkinson's disease, severe dementia, atrial fibrillation, coronary artery disease, coronary artery bypass surgery, permanent pacemaker placement, mitral valve replacement, hypertension, and glaucoma. The patient's oral intake decreased significantly over a two-week period. During the initial assessment, patient was alert, not oriented to person, place, or time, and confused. Her neurological exam was also significant for reactive aphasia and inability to follow commands. Vital signs were stable. Physical exam showed cachexia, no acute distress, bilateral air entry, and soft nontender abdomen with normal bowel sounds. No edema or bruising was noted on skin examination. Patient exhibited full range of motion. On the second day of admission, patient failed a dysphagia screening and began receiving fluid hydration intravenously. A 5% dextrose-0.45% saline infusion was started at 10 p.m. that evening at a rate of 100 mL/hour. The IV was checked at midnight, and the infusion rate was maintained. At 4 a.m., nursing staff noticed IV line infiltration. The patient was then found to have large blisters on the ventral side of her forearm. The IV line was immediately removed. On exam, serous blistering of various sizes distributed across the skin of the affected right forearm and significant swelling of the soft tissue was noted. A rigid nursing home identification bracelet proximal to midforearm was identified that was restricting the swelling to the distal forearm (Figure 1). Her skin was tense from the tips of her fingers to the midforearm with swelling visible in her knuckles as well as palmar and volar surfaces of the hand. Patient's sensory exam was indeterminate due to her baseline dementia. Her radial pulse was palpable. Surgical team was notified for suspected ACS of the forearm and the compartment pressures were measured with a Stryker (Kalamazoo, MI) as per the manufacturer's instructions. Superior and inferior forearm compartment pressures were between 20 mm Hg and 25 mm Hg; lateral and medial volar compartment pressures ranged between 50 mm Hg and 55 mm Hg on serial measurements. Given the measurements, diagnosis of ACS of the right hand and forearm was confirmed. Lactic acid, myoglobinuria, and kidney function were assessed as complications of ACS. All were within normal limits suggesting that no acute kidney injury or damage to muscle had occurred. The patient underwent a decompressive fasciotomy of the right hand and forearm with carpal tunnel release without complications. The fasciotomy immediately softened the hand and improved the motion of the joints. After the surgery, the patient was treated with prophylactic antibiotics and the dressing was changed daily with silver dressing. Seven days after the surgery, irrigation, excision, and debridement closure of forearm wound were performed. A PEG tube was placed on day 20 of admission to facilitate feeding. Patient's neurologic and vascular function of the affected limb remained intact after surgery.

## 3. Discussion

Normal resting muscular pressure is 0–8 mm Hg [6]. A review of the literature reveals that most compartment syndrome cases have pressures greater than 30 mm Hg. Once pressure reaches this level, capillary pressure is no longer sufficient to maintain microcirculation resulting in ischemic nerve damage [1, 4]. While there is no definitive threshold of compartment pressure requiring surgical decompression, most clinicians use a compartment pressure greater than 30 mm Hg as an indication for decompression in order to maintain blood flow and prevent complications [6]. In our patient superior and inferior forearm compartment pressures were between 20 mm Hg and 25 mm Hg while lateral and medial volar compartment pressures ranged between 50 mm Hg and 55 mm Hg making her an excellent candidate for surgical intervention. We believe that a pressure greater than 25 mm Hg should be used for indication of fasciotomy [2]. IV infiltration is a common problem during infusion treatments and can lead to rare complications like compartment syndrome. Signs and symptoms of acute compartment syndrome include pain, paresthesia (especially loss of two-point discrimination), pallor, pulselessness, and restricted mobility of the affected limb [6]. Diagnosis of compartment syndrome is based on clinical signs and symptoms. Pediatric patients, obtunded patients, and patients under anesthesia are unable to localize and express pain. These patients represent a high risk population for IV infiltration. Determination of pain, paresthesia, and paralysis are challenging in these groups [2, 4, 7]. Early signs and symptoms in such populations can be masked and recognition of IV infiltration may be delayed resulting in serious complications such as loss of function or amputation of the effected limb [1, 2, 7, 8]. In serious cases, infiltration may present as bullous skin lesions [5, 7, 9, 10], as we have presented here. In such patients compartment pressure measurement is recommended to help aid diagnosis [3]. Important complications in ACS include muscle damage and acute kidney injury. Lactic acid and myoglobinuria can be monitored to assess muscle breakdown and BUN and creatinine should be monitored for kidney function. Given our patients inability to express her pain and the obstruction caused by her identification bracelet her IV line infiltration resulted in her ACS. Acute compartment syndrome treatment requires emergent decompression within 6 hours to reduce potential morbidity [2]. Fasciotomy prevents ischemic nerve dysfunction by alleviating the tissue pressure and tissue perfusion [1, 5]. The number and location of incisions are determined by clinical findings and compartment pressures [2]. While some clinicians elevate the impacted limb as a temporary measure to decrease the pressure, this has no proven effect in decreasing pressure within the compartment [3]. In our case, fasciotomy of her hand and forearm with carpal tunnel release was done within four hours preventing further neurologic and vascular complications.

## 4. Conclusion

Acute compartment syndrome is a rare complication of IV infiltration. If it is not treated at early stage ACS can result in permanent damage to nerves and vessels. Infusion pumps are not designed for sensing IV infiltration. Thus, compartment syndrome may happen due to IV infiltration despite the use of properly functioning IV infusion pumps [3, 4, 8]. Our case suggests that, to prevent complications of IV infiltration injury, IV lines should be placed proximal to arm bands or bracelets and checked frequently. This is especially important in high-risk populations such as pediatrics, patients with altered mental status, anesthetized, and obtunded patients. Regular surveillance of IV lines in conjunction with proximal placement of lines to patient identification bracelets will lead to increased prevention of IV line infiltration and its complications.
